# AN UNUSUAL LOCATION OF BASAL CELL CARCINOMA: THE CLITORIS AND THE VULVA

**DOI:** 10.4103/0019-5154.44795

**Published:** 2008

**Authors:** Cömert Asuman, Akin Özlem, Tümerdem Burçak, Peker Önder

**Affiliations:** *From the Maltepe University School of Medicine, Department of Dermatology, Maltepe, İstanbul, Turkey*; 1 *From the Maltepe University School of Medicine, Department of Plastic, Reconstructive and Aesthetic Surgery, Maltepe, İstanbul, Turkey*; 2 *From the Maltepe University School of Medicine, Oruç Laboratory of Pathology, Maltepe, İstanbul, Turkey*

**Keywords:** *Basal cell carcinoma*, *clitoris*, *vulva*

## Abstract

Vulvar basal cell carcinoma (BCC) is rare, accounting for less than 5% of all vulvar neoplasms and less than 1% of all BCCs. Vulvar BCCs are usually diagnosed late because they are often asymptomatic and tend to grow at slow rates. They may be invasive and destructive if neglected or improperly treated. Nevertheless, they have a very low propensity for metastatic spread, but frequently recur after simple excision. We report a 78 year-old woman presenting with the complaint of painful vulvar ulceration and vaginal bleeding. The physical examination revealed a 3 × 2 cm indurated nodulo-ulcerative lesion involving the clitoris, both labia minora and left labium majus. The histopathology was consistent with the “solid type BCC” that invaded the subcutaneous tissue without lymph node metastasis. The patient underwent wide local excision with clitoral amputation and remained disease free at post-surgical follow-up after 18 months.

## Introduction

Basal cell carcinoma (BCC) is the most common malignancy of the skin, accounting for approximately 70–80% of all cutaneous cancers.^1^ The lifetime ultraviolet radiation damage is the most important factor in its pathogenesis, and the vast majority is observed on sun exposed skin, with nearly 85% occurring in the head and neck.^2^ Although BCCs can develop in sun protected areas, genital involvement is very rare, accounting for fewer than 1% of all cases.^3^ BCC accounts for 2–4% of all vulvar cancers (Table 1) and occurs most commonly in post-menopausal women.^4^ Since its first description by Temesvary in 1926, 200 cases of vulvar BCC have been listed in the literature.^5^ The etiology of vulvar BCC is unknown. Syphilis, chronic irritation, chronic infection, trauma, arsenicals, and radiotherapy have been implicated as possible precipitating factors.^5^,^6^ Clinically, vulvar BCC is an indolent and destructive tumor that rarely metastases, but the local recurrence rate is as high as 20% in some series.^5^,^6^

**Table 1:** Vulvar cancers

A.	Squamous-cell cancers (90%)
B.	Nonsquamous cancers (10%)
•	Bartholin's gland carcinoma
•	Malignant melanoma
•	Verrucous carcinoma
•	Paget's disease
•	Basal cell carcinoma
•	Sarcoma
•	Dermatofibrosarcoma protuberans
•	Kaposi's sarcoma
•	Metastatic malignant disease
•	Adenosquamous carcinoma
•	Lymphoma of the vulva
•	Merkel cell cancer (small)
•	Malignant fibrous histiocytoma

In this paper, we report an unusual case of locally invasive BCC located at the clitoris extending to the labia minora and left labium majus of a 78-year-old woman treated with wide local excision.

## Case Report

**Fig. 1:**
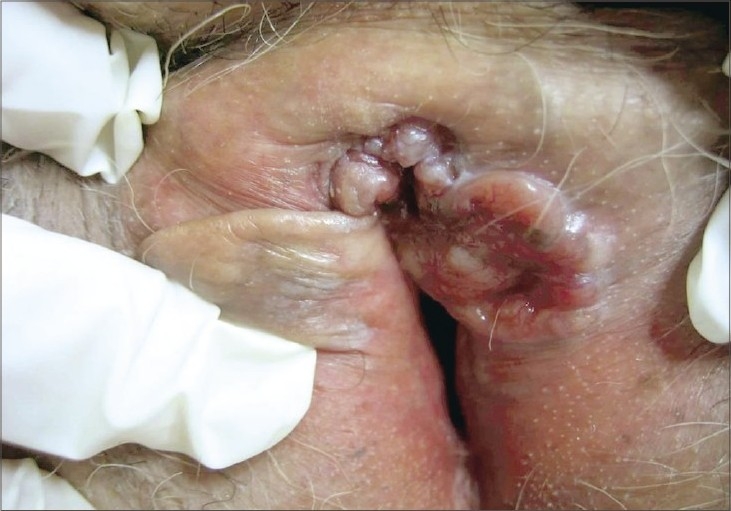
Multiple indurated nodules on the clitoris and an indurated ulcerous lesion of 3 × 2 cm size with an elevated telangiectatic border on the labia

**Fig. 2:**
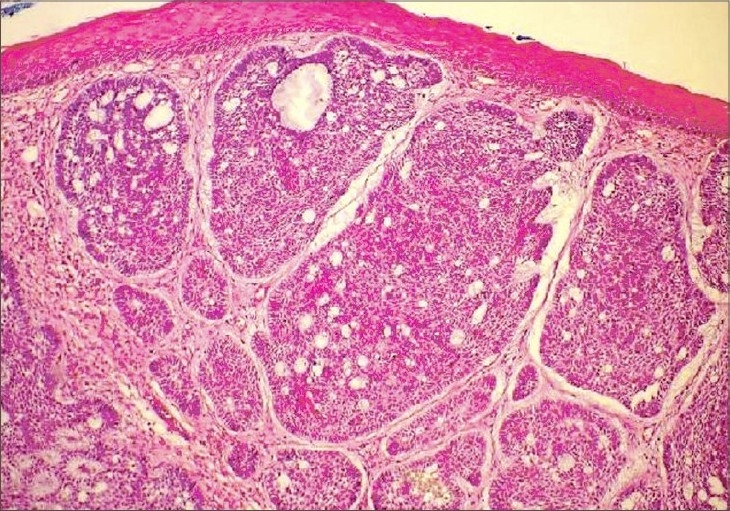
Dermal tumor masses composed of basaloid cells, peripheral palisading of the tumor nuclei and cleft formation (HE ×40)

A 78-year-old female presented with complaints of painful vulvar ulceration and bleeding for at least 4 months. She had been evaluated first by the gynecologist and then she was referred to us. She did not have a history of an antecedent skin disease or local irritation. She was hypertensive and underwent a total abdominal hysterectomy because of the intramural leiomyomas 6 months ago. She denied any personal or family history of skin cancers or internal malignancies. There was no history of sexually transmitted disease, irradiation or tobacco use. Physical examination revealed multiple indurated nodular lesions on the clitoris and a highly indurated ulcerous lesion (3 × 2 cm) with an elevated telangiectatic border on the labium majus (Fig. 1). The left labium minus, the superior portion of the right labium minus, medial part of the left labium majus and clitoris were completely eroded. Inguinal lymph nodes were not palpable and pelvic computed tomography verified the physical examination. An incisional biopsy was performed. Histopathologic examination revealed dermal aggregates of atypical basaloid cells with peripheral palisading and cleft formation consistent with the diagnosis of “solid type BCC” (Fig. 2). The patient underwent wide local excision of the tumor with the amputation of the clitoris. The surgical defect was reconstructed with bilateral advancement skin flaps. The pathologic examination of the excisional biopsy revealed solid-type BCC with the invasion of subcutaneous tissue. The lateral and deep excisional margins were all free of the tumor. There has been no tumor recurrence at the post-surgical follow-up after 18 months.

## Discussion

Vulvar BCC is a rare malignancy accounting for approximately 2–3% of all vulvar neoplasms.^2^,^3^,^5^ It usually affects white women over 70 years of age.^3^,^6^ Vulvar BCC may manifest itself as any clinical type of BCC. It usually presents as a nodule or like an ulcer in our case, but it may also have a very nonspecific and indolent clinical appearance. It may mimic other dermatological pathologies such as eczema, psoriasis, seborrheic keratosis or angiokeratoma.^2^ Therefore, it is recommended that all suspicious vulvar lesions should be biopsied for early diagnosis. The previous studies demonstrated that the tumor size ranged between 0.2 and 10 cm and most occurred on the labium majus and less commonly on the labium minus, urethral meatus, prepuce and clitoris.^5^–^7^ In our case, three parts of the vulva were invaded simultaneously by the tumor. The etiology of BCC in sun-protected areas remains unknown. The factors other than ultraviolet radiation seem to be involved. The literature suggests that radiotherapy to the pelvic region, chronic pruritus vulvae or ani, chronic vulvovaginitis, previous trauma such as burn or scar, arsenic, certain genetic conditions such as nevoid basal cell carcinoma syndrome and xeroderma pigmentosum, immune deficiency, human papillomavirus (HPV) infection (more relevant in squamous cell carcinoma of the genitalia), mutations in the p53 gene and advancing age may all contribute to the development of BCC in these sites.^2^,^3^,^5^–^7^ Although we could not search for the presence of HPV DNA, the advanced age seemed to play a contributor role in our patient. Although local excision is usually curative, recurrence and rare metastases have been reported, particularly in cases of the sclerosing type and those with perineural invasion.^3^,^4^ Our case had a nodular type of BCC and perineural invasion and lymphatic involvement were not present. Because of the tendency to be locally invasive and recurrent, wide surgical excision or Mohs micrographic excision are the recommended therapies for vulvar BCC.^6^–^8^ One centimeter margins seem to be adequate, particularly at the given age and general condition of the patient.^4^ Selective lymphadenectomy is warranted for the large invasive tumors with lymphatic involvement.^6^,^8^ In the case of incomplete excision or when surgery is contraindicated, radiation therapy is an alternative but often leads to local complications.^5^,^8^ Our patient underwent a wide local excision of the tumor and reconstruction with advancement skin flaps. At the post-surgical follow-up after 18 months, she is still tumor free. However, because of a high local recurrence rate of vulvar BCCs, close long-term follow-up is necessary.
